# Sterol O-Acyltransferase 2 Contributes to the Yolk Cholesterol Trafficking during Zebrafish Embryogenesis

**DOI:** 10.1371/journal.pone.0167644

**Published:** 2016-12-09

**Authors:** Nai-Yun Chang, Yen-Ju Chan, Shih-Torng Ding, Yen-Hua Lee, Wei-Chun HuangFu, I-Hsuan Liu

**Affiliations:** 1 Department of Animal Science and Technology, National Taiwan University, Taipei, Taiwan; 2 Institute of Biotechnology, National Taiwan University, Taipei, Taiwan; 3 The Ph.D. Program for Cancer Biology and Drug Discovery, College of Medical Science and Technology, Taipei Medical University, Taipei, Taiwan; 4 Research Center for Developmental Biology and Regenerative Medicine, National Taiwan University, Taipei, Taiwan; Oregon State University, UNITED STATES

## Abstract

To elucidate whether Sterol O-acyltransferase (Soat) mediates the absorption and transportation of yolk lipids to the developing embryo, zebrafish *soat1* and *soat2* were cloned and studied. In the adult zebrafish, *soat1* was detected ubiquitously while *soat2* mRNA was detected specifically in the liver, intestine, brain and testis. Whole mount *in situ* hybridization demonstrated that both *soat1* and *soat2* expressed in the yolk syncytial layer, hatching gland and developing cardiovascular as well as digestive systems, suggesting that Soats may play important roles in the lipid trafficking and utilization during embryonic development. The enzymatic activity of zebrafish Soat2 was confirmed by Oil Red O staining in the HEK293 cells overexpressing this gene, and could be quenched by Soat2 inhibitor Pyripyropene A (PPPA). The zebrafish embryos injected with PPPA or morpholino oligo against *soat2* in the yolk showed significantly larger yolk when compared with wild-type embryos, especially at 72 hpf, indicating a slower rate of yolk consumption. Our result indicated that zebrafish Soat2 is catalytically active in synthesizing cholesteryl esters and contributes to the yolk cholesterol trafficking during zebrafish embryogenesis.

## Introduction

Cholesterol is a multi-functional molecule and plays an important role in living organisms. It is one of the basic components in cell membrane, in which it modulates the integrity and fluidity, and serves as a structural component of lipid raft. It is also a precursor molecule for the synthesis of steroid hormones and bile acids. In addition, cholesterol is essential for the optimal neurotransmitter release, synaptogenesis, sonic hedgehog signaling and myelination [1]. Therefore, cholesterol deficiency during embryogenesis can lead to multiple embryonic abnormalities.

In the body, cholesterol can be acquired from two main sources: *de novo* synthesis and dietary intake. The *de novo* synthesis of cholesterol is tightly regulated at the transcriptional level through a negative feedback control correlated to the intracellular level of cholesterol. The cellular cholesterol level is detected by SREBP cleavage-activating protein (SCAP), which escorts Sterol Regulatory Element Binding Proteins (SREBPs) from endoplasmic reticulum (ER) to Golgi for proteolytic processing when cellular cholesterol falls below a critical level. The processed SREBP fragments translocate to the nucleus and initiate the transcription of target genes that involve in *de novo* cholesterol biosynthesis such as HMG-CoA reductase, a rate-limiting enzyme for cholesterol biosynthesis [2, 3]. On the other hand, an efficient absorption of dietary cholesterol and the transportation of free cholesterol in the body require the esterification of cholesterol by Sterol O-acyltransferase (SOAT) before these lipid molecules are packaged into chylomicrons [4].

Soats, also known as acyl-CoA: cholesterol acyltransferases or briefly ACATs, catalyze the esterification of cholesterol with long chain fatty acyl-CoA to form cholesteryl esters (CEs) at ER membrane. Soats are the founding members of the membrane-bound O-acyl-transferase (MBOAT) family, which are multispan membrane proteins that transfer fatty acyl groups onto hydroxyl groups of their targets such as cholesterol, glycerol and sugars. In mammals, there are two isoforms of SOAT, SOAT1 and SOAT2, which share about 50% amino acid sequence homology in human and mice [5, 6] and are highly similar near the COOH-terminus [7, 8]. However, the distinct expression profiles [9, 10] and potentially different membrane topologies [11, 12] of SOAT1 and SOAT2 led to the general belief that SOAT1 plays its role in the maintenance of intracellular cholesterol homeostasis, while SOAT2 mediates the absorption and transportation of dietary cholesterol. Nevertheless, both mammalian SOAT1 and SOAT2 are capable of synthesizing CEs, which can lead to the accumulation of intracellular lipid droplets [13] and can contribute to the lipid core in lipoproteins [14, 15].

For oviparous animals, the embryos are limited to the resource within the eggs, which usually contain large reserves of lipid droplets in their yolks. During the first four days of development, zebrafish embryos are lecithotrophic and rely on the yolk syncytial layer (YSL) to transport yolk nutrients to the developing embryos [16]. Previous studies showed that apolipoproteins and microsomal triglyceride transfer protein are expressed in YSL [17–19]. Furthermore, defective lipoprotein assembly during embryogenesis resulted in an unabsorbed yolk phenotype [19, 20] suggesting that yolk lipids were assembled into lipoproteins at YSL for the delivery to the developing embryos. Since Soats play a role in cholesterol esterification and in turn lipoprotein assembly, it is reasonable to hypothesize that they might be responsible for converting yolk cholesterol and fatty acyl groups into CEs, and in turn contribute to the transportation of yolk lipids to the zebrafish embryo. In this study, we cloned and characterized zebrafish *soat2* for its enzymatic activity, and profiled its temporal and spatial expression patterns during early zebrafish embryogenesis. Furthermore, we used zebrafish to investigate the role of Soat2 in the transportation of yolk lipids during early embryonic development.

## Materials and Methods

The AB strain wild-type zebrafish were housed at a density of 2–4 fish per 3-L tank in the aquatic facility with an automatic recirculation system. The system was maintained at 28.5°C with a light/dark cycle of 14/10 h, and the fish were fed with live adult brine shrimp twice a day [21]. Embryos were collected after spontaneous spawning, allowed to develop and staged by hour-postfertilization (hpf) at 28.5°C using morphological criteria [22]. All experimental procedures in this study were reviewed and approved by the Institutional Animal Care and Use Committee (IACUC) of National Taiwan University (NTU-100-EL-73) and were performed in accordance with the approved guidelines.

### Molecular cloning and rapid amplification of cDNA ends

To extract the total RNA, zebrafish embryos were homogenized in 1mL TRIzol Reagent (Life Technologies, Carlsbad, CA, USA) and incubated at room temperature for 5 min. Homogenates were mixed with 100 μL of 1-Bromo-3-chloropropane and then centrifuged at 12,000 × *g* for 15 min at 4°C. The aqueous phase was transferred into a new tube and mixed with 500 μL of isopropanol. The mixture was incubated for 5 min and centrifuged at 12000 × *g* for 8 min at 4°C. The pellet was then washed with 1 mL of 75% ethanol and centrifuged at 7,500 × *g* for 5 min at 4°C. The ethanol was removed and the RNA pellet was briefly air-dried for 3–5 min. The resulted RNA was resuspended in DEPC-treated water and single-strand cDNA was synthesized from 1μg of total RNA with random primers and SuperScript III Reverse Transcriptase (Life Technologies).

To evaluate the expression profiles, 1 μL of target specific gene primers (Table 1), 1 μL of cDNA and 0.1 μL of DNA Taq polymerase (Life Technologies) were mixed in a total volume of 50 μL and the samples were placed in the thermal cycler. PCR was performed with the following program: 94°C for 2 min, then 30 cycles of 94°C for 20 sec, 60°C for 10 sec, 72°C for 40 sec and the final polymerization was performed at 72°C for 5 min. The sequences of the primers used in this study were listed in Table 1.

**Table 1:** Sequences of the oligos used in this study.

Name	Sequence (5’- … -3’)
*For expression profiling*	
soat1-F	AGCAGACCTGATGAGGCAGT
soat1-R	CCAAACGCATACACCAACAG
soat2-F	TTCTTGTTCTGCCCGACTCT
soat2-R	CAAAGCCTGATCCGTCCTG
β-actin-F	ATGGATGAGGAAATCGCTGCCCTGGTC
β -actin-R	CTCCCTGATGTCTGGGTCGTCAAC
*For overexpression*	
soat1-F-all	GAACAACACACGGCAGCA
soat1-R-all	CGTTCCACATTCTGTTACGG
soat2-F-all	CAGGACGGTGATCAGAGAATC
soat2-R-all	AGCACACAGTCGGTTAGTGAT
*For RACE*	
Soat1-5RACE	GCTCCACCTCCACTTTTCC
Soat1-5RACE-nest	GCGTCTCTTTCAGGTCCATC
Soat1-3RACE	GGACCGATCTGGAACGTG
Soat1-3RACE-nest	GGACAGGGGGTAATGATCT
Soat2-5RACE	CTCCACCGCATCATTCAG
Soat2-5RACE-nest	GGTCCTGATGTGGCTGATCT
Soat2-3RACE	TTCAGGACGGATCAGGCTTT
*For riboprobe preparation*	
soat1-probe-F	TGTTCTGCCTCTTCATGTGC
T7-soat1-probe-R	actcactatagggagatgACATCACAAAGGCGTCTAA
soat2-probe-F	GCTGTTTGAGATCAGCCACA
T7-soat2-probe-R	actcactatagggagatgCTGTGGATTCACGGATAA
*For overexpression in HEK293*	
soat1-F-all	GAACAACACACGGCAGCA
soat1-R-all	CGTTCCACATTCTGTTACGG
soat2-F-all	CAGGACGGTGATCAGAGAATC
soat2-R-all	AGCACACAGTCGGTTAGTGAT
*For validation of HEK293 transfection*	
eGFP-F	ACGTAAACGGCCACAAGTTC
eGFP-R	AGTTCACCTTGATGCCGTTC
soat1-F	AGCAGACCTGATGAGGCAGT
soat1-R	CCAAACGCATACACCAACAG
soat2-F	ATGCGGTGGAGCGCGCCGTT
soat2-R	CCCTGGTCGATGTAGTCCAC
*For morpholino characterization*	
s2-MO	TCAGCCTCTGAAGACAAACATTCGT
Soat2-e3-F	GAATGGCAGGAATATTACAGAGG
Soat2-e6-R	CCCTGGTCGATGTAGTCCAC

To clone the full length coding sequences of *soat1* and *soat2*, specific primers for *soats* (10 mM) (Table 1), PfuUltra II HotStart DNA Polymerases (Agilent, Santa Clara, CA, USA), and 1 μL of cDNA were mixed for PCR reaction and the products were purified using QIAquick PCR Purification Kit (Qiagen, Hilden, Germany). After PCR products were purified, the samples were ligated into the pGEM-T EASY vectors (Promega, Madison, WI, USA). The coding regions were confirmed by sequencing service (Center for Biotechnology, National Taiwan University, Taipei, Taiwan).

To confirm the complete sequences of *soat1* and *soat2*, rapid amplifications of 5’- and 3’-cDNA ends (5'-RACE and 3'-RACE) were performed using GeneRacer Kit (Life Technologies) according to the manufacturer’s instruction. Briefly, total RNA was extracted from zebrafish embryos (24 hpf) and 5’- and 3’-cDNA ends of *soat*s were amplified by using PfuUltra II HotStart DNA Polymerases (Agilent), specific primers (Table 1) and GeneRacer Primers. PCR was performed by the following program: 95°C for 1 min, then 30 cycles of 95°C for 20 sec, 57°C for 10 sec, 72°C for 90 sec followed by final polymerization at 72°C for 3 min. The PCR products were purified, ligated into the pGEM-T EASY vectors (Promega) and sequenced (Center for Biotechnology).

To express Soat enzymes, the coding sequences of *soats* were subcloned into the pT7-IRES2-DsRed or pT7-IRES2-eGFP vectors [21] with *EcoR*I to generate pSoat1-IRES2-DsRed, pSoat1-IRES2-eGFP, pSoat2-IRES2-DsRed or pSoat2-IRES2-eGFP, respectively. These plasmids allow the cistronic expression of a fluorescent protein (eGFP or DsRed) together with the genes of interest and hence the expression could be confirmed under a fluorescent microscope.

### Whole-mount in situ hybridization

To demonstrate the spatial expressions of zebrafish *soats* during embryonic development, whole-mount *in situ* hybridization was performed as described previously [21]. Briefly, a 300~400 bp of sense and antisense digoxigenin-labeled riboprobes for *soat2* were synthesized by *in vitro* transcription using T7 polymerase with the T7 RNA polymerase promoter introduced at the 5’-end of the probe sequences by performing nested PCR. Zebrafish embryos were dechorionated, fixed with 4% paraformaldehyde (PFA) in PBS, and digested with proteinase K if older than 24 hpf. The embryos were then pre-hybridized for 3 hrs at 65°C without riboprobes and then hybridized with 50 ng RNA probe at 65°C water bath overnight. After washing, hybridized embryos were blocked for 3 hrs at room temperature and incubated with Anti-Digoxigenin-AP Fab fragments (1:5000 in blocking solution; Roche Applied Science, Mannheim, Germany) with agitation at 4°C overnight. After washing, the hybridization signals were detected by NBT/BCIP solution (Roche Applied Science), observed and documented with microscope (Leica Z16-APO).

### Quantitative analysis of intracellular lipid accumulation

To characterize the enzymatic activity of zebrafish Soat2, human embryonic kidney 293 (HEK293, 1×10^6^) cells were transfected with pSoat1-IRES2-eGFP, pSoat2-IRES2-eGFP or pIRES2-eGFP using TransIT-LT1 Transfection Reagent (Mirus Bio, Madison, WI, USA) according to the manufacturer’s instruction. After the green fluorescent signal was detected, the cells were maintained in Dulbecco’s modified Eagle’s medium/F12 (DMEM/F-12; Life Technologies) with 10% fetal bovine serum (FBS; Life Technologies) containing G418 (400 μg/mL) (AG Scientific, San Diego, CA, USA) for 2–3 weeks. The drug resistant colonies were isolated and maintained in 200 μg/mL of G418.

To quantify the overall intracellular neutral lipid, two days after initial seeding (1×10^5^ cells) into 24-well plates, HEK293 cells were incubated with or without Pyripyropene A (PPPA; 10 μg/ml in DMSO; Enzo Life Sciences, Farmingdale, NY, USA) or Avasimibe (AVA; 5 μg/mL in DMSO; Sigma-Aldrich, St. Louis, MO, USA) overnight, and then supplemented with 15 μM oleic acids (dissolved in DMSO) (Cayman Chemical, Ann Arbor, MI, USA) and 1 μg/mL of cholesterol (dissolved in ethanol) (Sigma-Aldrich), or with 150 μM oleic acids and 10 μg/ml of cholesterol at room temperature for 6 hrs. The medium was then removed and the cells were gently rinsed with PBS. The cells were then fixed with 10% formaldehyde at room temperature for 15 min, rinsed with water for 3 times, and incubated in 1 mL of 100% propylene glycol for 2 min. To detect the neutral lipid in the cells, the cells were incubated with Oil Red O (ORO) for 15 min, rinsed in 60% propylene glycol for 1 min and washed with water twice. Finally, to quantitatively evaluate the ORO trapped in the cells, 200 μL of DMSO were added per well and the absorbance was measured at 550 nm. The total amount of ORO in each well was standardized to the ORO staining solution within the linear range of absorbance.

To specifically quantify intracellular CEs, a colorimetric assay (K623-100, BioVision, Milpitas, CA, USA) was used following the instructions of the manufacturer. Briefly, after seeding 1×10^6^ cells into 6-well plates, HEK293 cells were incubated for 24 hrs, and then supplemented with 150 μM oleic acids (dissolved in ethanol) and 10 μg/ml of cholesterol (dissolved in ethanol) at 28.5°C temperature for 6 hrs. The cells were then counted using a hemocytometer for data normalization, homogenized in 200 μL of lysis buffer (CHCl_3_: isopropanol: NP-40 = 7:11:0.1), and centrifuged at 15,000 ×*g* for 5 min. The lipids containing liquid phase was dried for 1 hr under vacuum at 50°C. The dried lipids were then dissolved with 200 μL of Cholesterol Assay Buffer under sonication for 3 min and vortex for 30 sec. For every reaction, 45 μL of extract sample was adjusted to 50 μL with 5 μL of Cholesterol Assay Buffer. To determine total cholesterol, 2μL Esterase was also added into each sample with 30 min incubation at 37°C. To assess the amount of free cholesterol, the reaction mix (2μL of Substrate Mix, 2μL of Cholesterol Enzyme Mix and Cholesterol Assay Buffer) was added to each sample to a total 100 μL of reaction volume. The reaction was incubated at 37°C in the dark for 30 minutes. The quantities of free cholesterol and total cholesterol were acquired by measuring the light absorbance of samples at 450 nm in a micro-plate reader (SpectraMax190, Molecular Devices, Sunnyvale, CA, USA), calculated according to the standard curve and normalized to the cell count. The quantity of cholesteryl ester was determined by subtracting the value of free cholesterol from the value of total cholesterol. The average quantities of CE were calculated and presented as the results.

### Visualization of the cholesterol dynamics by NBD-cholesterol

To visualize the accumulation of cholesterol and CEs in the cells, HEK293 cells (1×10^5^) were seeded into 12-well dish in DMEM/F12 containing 10% FBS, and were transiently transfected with pSOAT2-IRES2-DsRed or pIRES2-DsRed using TransIT-LT1 Transfection Reagent. After the red fluorescent signal was detected, the transfected cells were incubated with 10 μg/mL of 22-(N-(7-Nitrobenz-2-Oxa-1,3-Diazol-4-yl)Amino)-23,24-Bisnor-5-Cholen-3β-ol (NBD-cholesterol; Life Technologies) at room temperature for 5 hrs. The cells were then fixed 4% PFA for 10 min and washed briefly with PBS. After incubated in DAPI staining solution for 3~5 min at room temperature, the cells were mounted on the glass slides with mounting medium and the fluorescent micrographs were documented using a Leica TCS SP5 II confocal microscope.

### Morphometric analysis for yolk cholesterol consumption

To manipulate the yolk Soat2 activity, PPPA (0.24 ng/embryo) AVA (1.4 ng/embryo), DMSO or morpholino oligos were injected (0.5 nL) into the yolk of 3~4 hpf embryos [23]. The *soat2* specific morpholino (s2-MO, 4.2 ng/embryo) was designed and purchased (Gene Tools LLC, Philomath, Oregon, USA). The sequences of the morpholino oligo and the primers for characterizing this morpholino oligo were as listed in Table 1. Since yolk injection could potentially harm the embryo, all the treated embryos with normal gross appearance were selected for following analysis. To estimate the yolk lipid consumption, the yolk size of individual embryo was estimated [19, 20]. Briefly, the embryos were photographed between 48 and 96 hpf, and the sizes of the yolks were measured using ImageJ software [24]. For each embryo, the result was the average of 3 measurements.

### Statistical analysis

To assess the intracellular lipid accumulation and yolk size in zebrafish emrbyos, one-way ANOVA with Tukey’s multiple comparisons test was used, while two-way ANOVA with Bonferroni’s multiple comparisons test was used to determine the statistical significance for the yolk sizes in yolk injection study. All statistical tests with P values < 0.05 were considered statistically significant. All data are reported as mean ± standard error of the mean both in the histograms and in the text.

## Results

### Zebrafish soats are conserved throughout evolution

**Fig 1:**
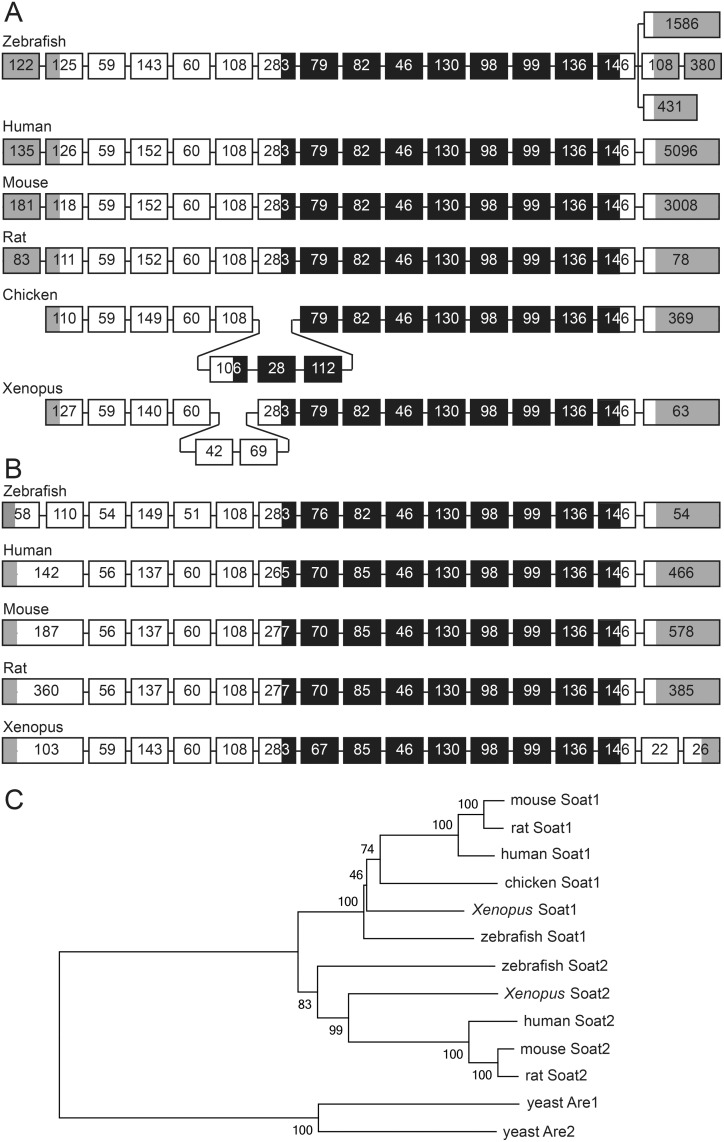
Comparative *in silico* analysis of soat1 and soat2. (A-B) Genomic organizations of *soat1* (A) and *soat2* (B) in zebrafish, human, mouse, rat, *Xenopus* and chicken showed similar pattern of exonal distributions. In the regions coding for MBOAT domains, the exon/intron structures are almost indentical between *soat1* and *soat2* and among various species. The sizes of the exons are shown in the box representing exons, the grey area in the exon boxes are untranslated regions, and the black regions represent MBOAT domains. (C) Phylogenetic trees were constructed using neighbor joining method with percentage identity distances with the software Jalview2, and showed that *soat1* and *soat2* genes are grouped together respectively, with yeast ARE1 and ARE2 as outgroups. The reference genes used in this comparative analysis include: *SOAT1* of human (ENST00000367619), mouse (ENSMUST00000051396), rat (ENSRNOT00000005677), chicken (ENSGALT00000006691), *Xenoupus* (ENSXETT00000050224), *SOAT2* of human (ENST00000301466), mouse (ENSMUST00000023806), rat (ENSRNOT00000015368), *xenopus* (ENSXETT00000003832), and yeast are1 (YCR048W), and are2 (YNR019W). The sequences of zebrafish *soat1* and *soat2* were obtained from the cloned sequences.

**Fig 2:**
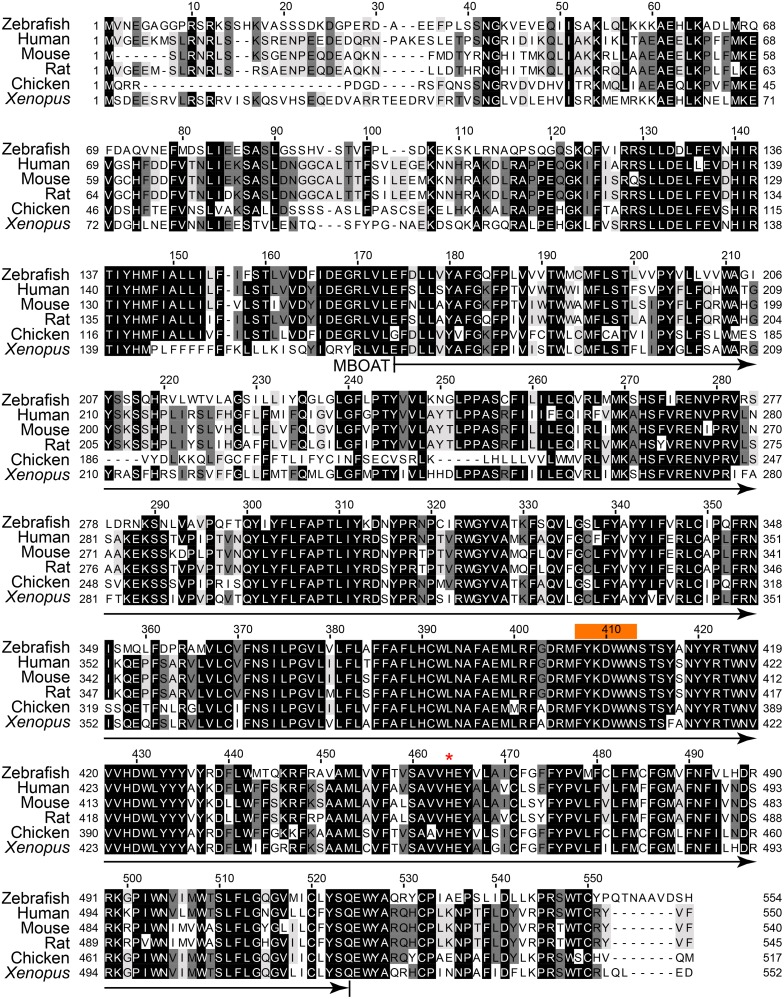
Sequence similarity of Soat1 among various animal species. Zebrafish Soat1 amino acid sequences are aligned with the SOAT1 orthologs from human (ENSP00000356591), rat (ENSRNOP00000005677), mouse (ENSMUSP00000058344), chicken (ENSGALP00000006691) and *Xenopus* (ENSXETP00000050224). The amino acid sequence of zebrafish Soat1 was predicted according to the cloned sequence. The MBOAT domain is predicted between 168 to 517 residue (arrow under sequences). The acyl-CoA binding domain (FYXDWWN, orange box above the sequences) and the postulated catalytical residue for esterification (His457, red asterike above the sequences) are conservative through species. The identical amino acids are shown in the black box, while the less conservative regions are enclosed by white box.

**Fig 3:**
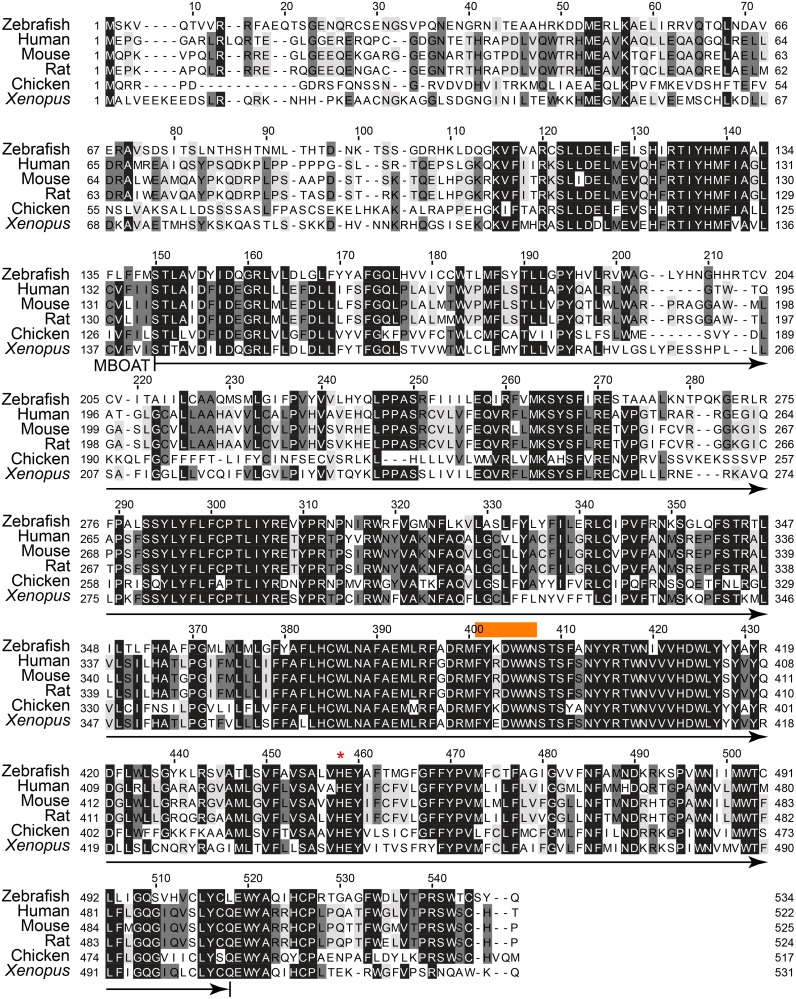
Sequence similarity of Soat2 among various animal species. Zebrafish Soat2 amino acid sequences are aligned with the SOAT2 orthologs from human (ENSP00000301466), rat (ENSRNOP00000015368), mouse (ENSMUSP00000023806), and *Xenopus* (ENSXETP00000003832). The amino acid sequence of zebrafish Soat2 was predicted according to the cloned sequence. The MBOAT domain is predicted between 140 to 505 residue (arrow under sequences). The acyl-CoA binding domain (FYXDWWN, orange box above the sequences) and the postulated catalytical residue for esterification (His445, red asterike above the sequences) are conservative through species. The identical amino acids are shown in the black box, while the less conservative regions are enclosed by white box.

To study *soat2* in zebrafish, we first *in silico* identified zebrafish *soat* genes and compared the genomic structure among different species including human, mouse, rat, chicken, and *Xenopus*. All the Soat orthologs contained a conserved enzymatic domain of MBOAT family and the structures of exons encoding MBOAT domain shared a high level of similarity among these species (Fig 1A). To obtain the full-length sequences of *soat* transcripts, rapid amplification of 5’- and 3’-cDNA ends (5’-RACE and 3’-RACE) were performed with total RNA extracted from 24 hpf embryos. The RACE results showed that *soat1* contained 3 different transcripts, which share the same 5'-untranslated region (5’-UTR) and coding sequences with different lengths of 3'-UTR (S1 Fig), while only one transcript of *soat2* was found in zebrafish (Fig 1B). The coding sequence of *soat1* was predicted to translate into 554 amino acids (Fig 2), while *soat2* was predicted to translate into 534 amino acids (Fig 3). Furthermore, the phylogenetic tree analysis using a neighbor joining method with percentage identity distances with the software Jalview2 [25] showed that zebrafish Soat1 and Soat2 are conserved throughout the evolutionary history (Fig 1C).

Aligning the translated Soat1 and Soat2 orthologs from different species including human, rat, mouse, *Xenopus* and chicken, we found that zebrafish Soat1 and Soat2 protein sequences were very similar to those compared species, especially in the MBOAT domain, the acyl-CoA binding domain [11, 26] (FYXDWWN, orange box in Figs 2 and 3), and the catalytically critical histidine residue [11, 27] (His457 in Soat1 and His445 in Soat2) (Figs 2 and 3). These results suggested that zebrafish Soat1 and Soat2 are highly conserved not only in the sequence but also their protein structure.

### Zebrafish soat2 is expressed in a temporal-spatial specific and tissue specific pattern

**Fig 4:**
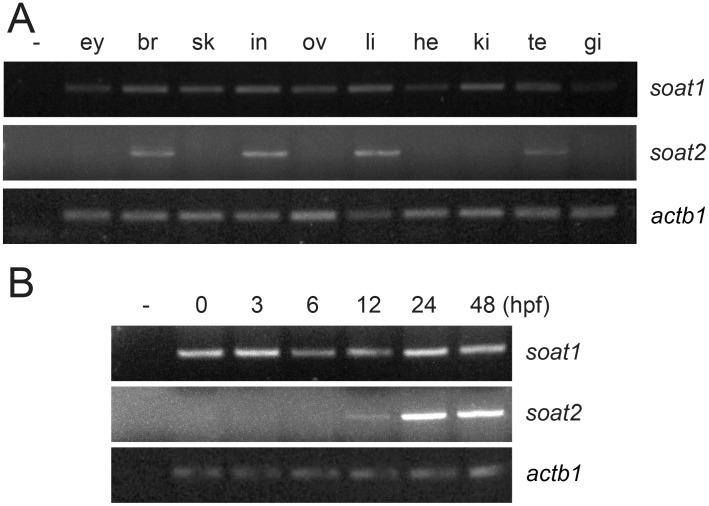
Distinct expression profiles of *soat1* and *soat2*. RT-PCR analysis using cDNA collected from zebrafish adult tissue (A) and embryos (B) indicated that *soat1* is expressed ubiquitously in every tissue tested, including the eye (ey), brain (br), skin (sk), intestine (in), ovary (ov), liver (li), heart (he), kidney (ki), testis (te) and gill (gi), and could be detected at freshly laid eggs (0 hpf) through 48 hpf. *Soat2* is expressed only in the brain, liver, intestine and testis in the adult zebrafish, and could not be detected until 12 hpf in the embryos. β-actin served as loading control while the reaction without template (-) served as negative control.

One of the important differences between mammalian Soat1 and Soat2 that might confer to their distinct physiological roles is their distinct expression patterns. We therefore performed RT-PCR with mRNA extracted from various organs of adult zebrafish and showed that *soat1* was expressed ubiquitously in tissues we examined, including the heart, intestine, liver, skin, ovary, testis, eyes, kidney, brain and gills, while *soat2* was expressed restrictively in the brain, liver, intestine and testis (Fig 4A). During the embryonic development, *soat1* expression can be detected in the freshly laid eggs (0 hpf) through 48 hpf, while the expression of *soat2* was not detected until 12 hpf and rapidly increased after 24 hpf (Fig 4B).

**Fig 5:**
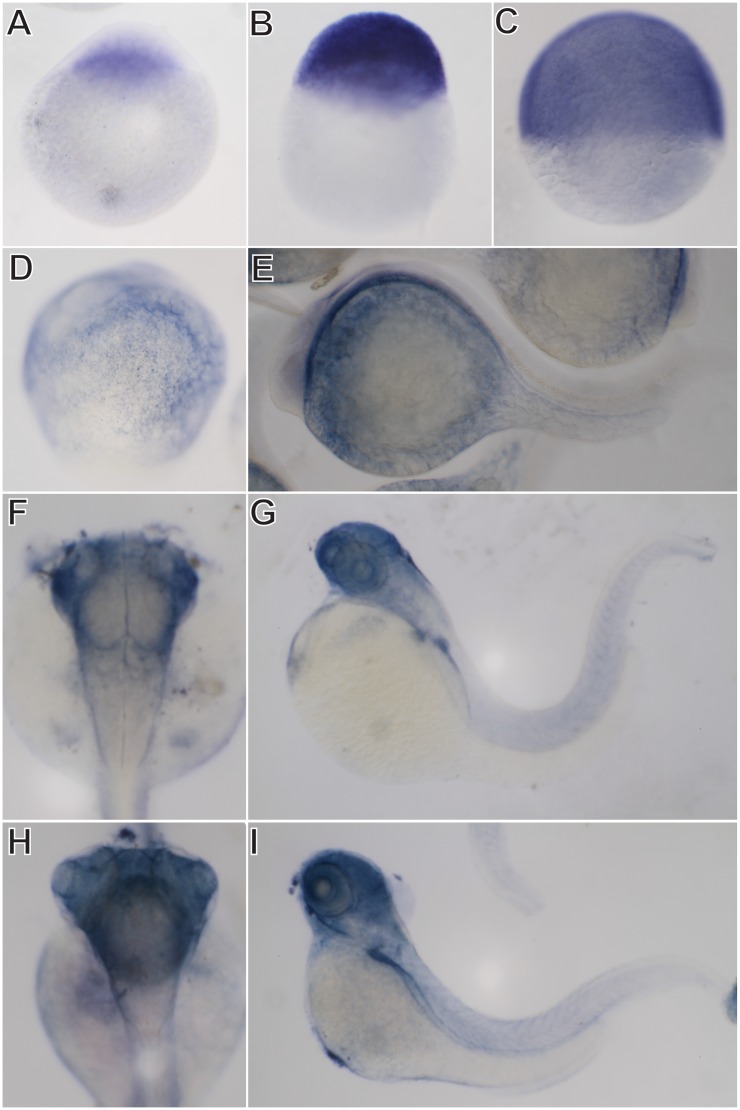
Expression patterns of zebrafish *soat1* during embryogenesis. Whole-mount *in situ* hybridization shows that *soat1* can be detected in all blastomeres at 1 cell stage (A), 3 hpf (B), and 6 hpf (C). The expression of *soat1* was prominent at yolk sac at 12 hpf (D) and 24 hpf (E). By 48 hpf (F, G), *soat1* was detected in the brain, retina, pectoral fin, hatching gland and pericardium. By 72 hpf (H, I), *soat1* was also observed prominently in the liver primordia and intestine.

**Fig 6:**
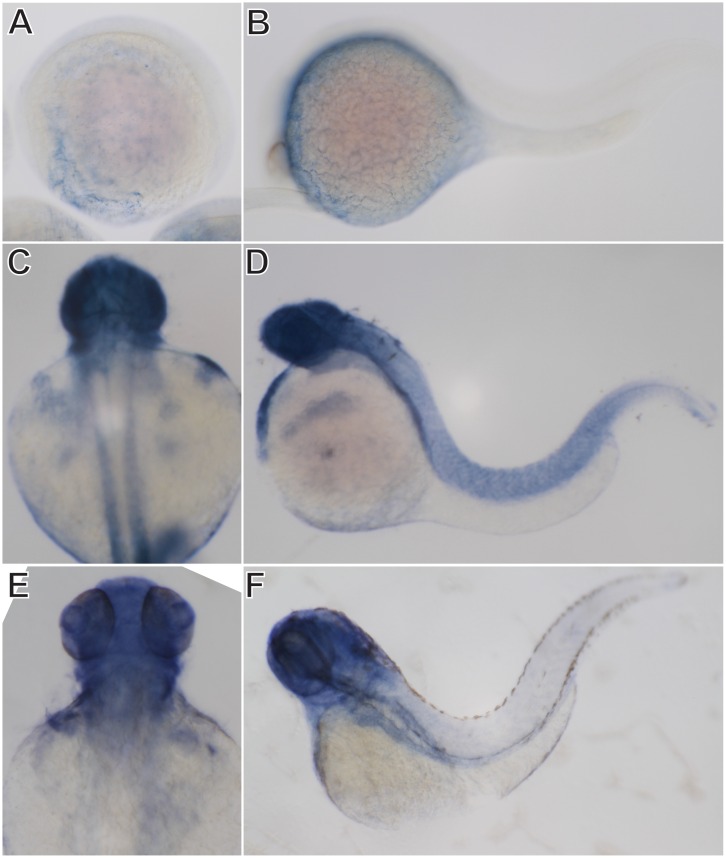
Expression patterns of zebrafish *soat2* during embryogenesis. Whole-mount *in situ* hybridization shows that *soat2* can be detected at yolk sac at 12 hpf (A) and 24 hpf (B). By 48 hpf (C, D), *soat2* was detected in the brain, retina, yolk sac, hatching gland, pericardium and intersomitic regions. By 72 hpf (E, F), *soat2* was also observed in the liver primordial and intestine.

To further depict the spatial expression patterns of *soat1* and *soat2*, whole-mount *in situ* hybridization was performed. In accordance with the result of RT-PCR, the expression of *soat1* was ubiquitously detected in all blastomeres at 1-cell stage, 3 hpf and 6 hpf (Fig 5A–5C). At segmentation period (12 hpf), *soat1* expression was located in the YSL (Fig 5D). At 24 hpf, *soat1* mRNA was detected in the central nervous systems, YSL and pharyngeal arches (Fig 5E). By 48 hpf, *saot1* mRNA can be detected in the brain, retina, pectoral fin, hatching gland and pericardium (Fig 5F and 5G). At 72 hpf, *soat1* transcripts were also observed in the brain, retina and primordial liver and intestine (Fig 5H and 5I). After 48 hpf, the expression of *soat1* in the intersomitic regions was also detected where the intersomitic vessels are developing (Fig 5G and 5I). The expression of *soat2* was observed in the yolk spheres at 12 and 24 hpf (Fig 6A and 6B). By 48 hpf, *soat2* transcripts were observed in the brain, retina, pectoral fin, heart and hatching gland (Fig 6C and 6D). At 72 hpf, the expression of *soat2* was located at brain, retina, heart and primordial liver and intestine (Fig 6E and 6F). The expressions of both *soat* genes in the YSL, hatching gland and partially in the developing cardiovascular and digestive systems through early embryonic development were noted.

### Overexpression of soat2 increased the intracellular lipid droplets

**Fig 7:**
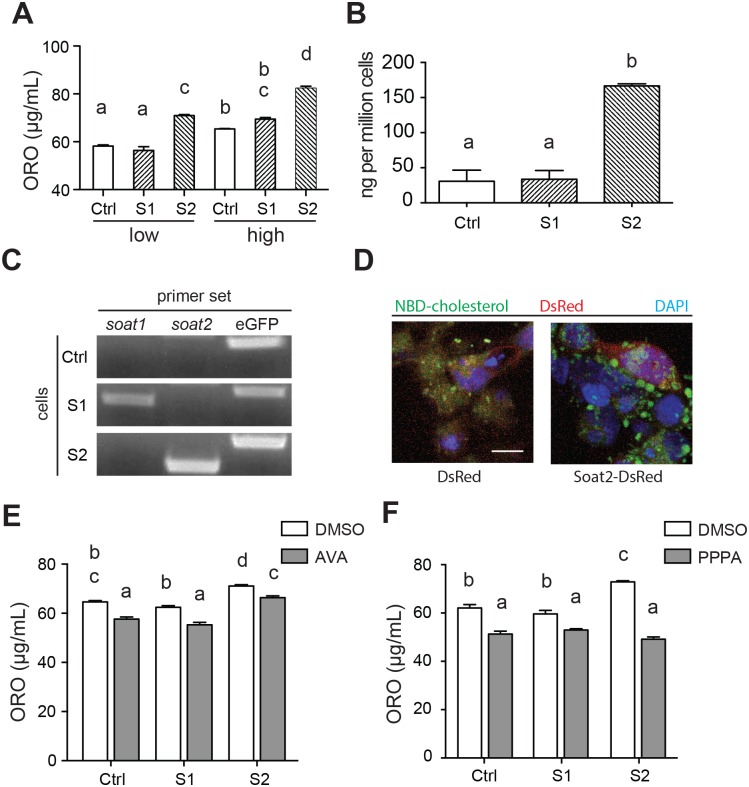
Increased intracellular accumulation of neutral lipid and CEs as a result of esterification of cholesterol to fatty acyl-CoA. (A) Oil Red O (ORO) staining of eGFP stable expressing (Ctrl), zebrafish *saot1* stable expressing (Soat1), and zebrafish *soat2* stable expressing (Soat2) HEK293 cells after incubation with low (15 μM oleic acids and 1 μg/mL cholesterol) or high (150 μM oleic acids and 10 μg/mL cholesterol) level of substrate at room temperature for 6 hrs. The expression of zebrafish Soat2 significantly increased the intracellular accumulation of neutral lipid indicated its enzymatic activity of cholesterol esterification, and this accumulation of neutral lipid is significantly increased with the increased levels of substrates. Note the significantly increased neutral lipid in WT-high compared to WT-low reflected the activity of endogenous human SOAT1 expressed by HEK293 cells. The letters above each column indicate the statistical groups, and the data sharing the same letters indicates no significant difference. (B) The cell lysate of HEK293 cells with zebrafish *soat2* overexpression contained significantly higher CE content than either control group or *soat1* overexpression group. Consistent with the ORO staining, the CE content in *soat1* group was comparable to the control group. (C) RT-PCR confirmed the expression of both zebrafish *soat1* and eGFP in Soat1-overexpressing HEK293 cells, zebrafish *soat2* and eGFP in Soat2-overexpressing HEK293 cells and only eGFP in control group in which the HEK293 cells were transfect with empty vectors. (D) The HEK293 cells overexpressing zebrafish Soat2 (Soat2-DsRed) contained more and larger intracellular lipid droplets containing NBD-cholesterol as compared to the HEK293 cells overexpressing DsRed only (DsRed). (E) Avasimibe (AVA) significantly reduced the intracellular accumulation of neutral lipid, but the cells with zebrafish *soat2* overexpression still showed a significantly more ORO staining than other AVA-treated cells. (F) Pyripyropene A (PPPA) could significantly reduce the intracellular accumulation of neutral lipid by inhibiting the enzymatic activity of both endogenous human SOAT1 and zebrafish Soat2 when compared to the vehicle control (DMSO). The letters above each column indicate the statistical groups, and the data sharing the same letters indicates no significant difference.

To confirm whether zebrafish Soats could catalyze cholesterol esterification thereby increasing intracellular neutral lipids, HEK293 cells stably expressing eGFP, zebrafish Soat1 and Soat2 were incubated with oleic acids and cholesterol at room temperature for 6 hrs and the amount of intracellular neutral lipids were evaluated by ORO staining. The accumulated neutral lipids in Soat2-expressing cells were significantly higher than eGFP- and Soat1-expressing HEK293 (P<0.001, *n* = 3) (Fig 7A). Interestingly, we observed significantly increased neutral lipids with the increased supplementation of cholesterol and oleic acid in all groups (WT, low vs. high, P<0.001, *n* = 3; Soat1, low vs. High, P<0.0001, *n* = 3; Soat2, low vs. high, P<0.0001, *n* = 3) with Soat2-expressing cells significantly higher than both wild-type and Soat1-expressing HEK293 (P<0.001, *n* = 3) (Fig 7A). To specifically evaluate the enzymatic activity of Soats, the amount of cholesterol were quantified using a colorimetric assay with or without the pre-treatment of cholesterol esterase so that the exact amount of CEs could be specifically estimated. Consistent with the results of ORO staining, the accumulated CEs in Soat2-expressing cells were significantly higher than eGFP- and Soat1-expressing HEK293 (P<0.0005, *n* = 3), while no difference was detected between eGFP- and Soat1-expressing HEK293 (Fig 7B). All the cells were indeed expressing our genes of interest as indicated by RT-PCR (Fig 7C). A similar result was observed under confocal microscope after adding fluorescent NBD-cholesterol to HEK293 transiently overexpressing DsRed, Soat1-DsRed or Soat2-DsRed. Only the Soat2-DsRed expressing cells contained more and bigger NBD-cholesterol labeled intracellular lipid droplets compared to the cells transfected with empty vector (Fig 7D and S2 Fig).

To further confirm the increased intracellular lipid droplets was due to the overexpressed zebrafish Soat2 in HEK293, we measured intracellular accumulation of neutral lipids with the cells pretreated with Soat inhibitor, PPPA or AVA. PPPA has much stronger inhibitory effect against Soat2 then it against Soat1, while AVA has been considered non-selective inhibitor against both Soat1 and Soat2 in mammalian cells [28]. Previous reports showed that AVA could inhibit CE synthesis conducted by SOAT1 in human macrophage in the cell culture at the concentration of 0.05~1 μg/mL [29, 30]. Accordingly, the treatment of 5 μg/mL AVA resulted in a reduction in total neutral lipids when compared to the vehicle control within each gene-group (Ctrl: DMSO vs. AVA, P<0.001; S1: DMSO vs. AVA, P<0.001; S2: DMSO vs. AVA, P<0.01) (Fig 7E) indicating the activity of endogenous human SOAT1 was indeed inhibited. However, AVA treated cells with zebrafish soat2 overexpression still showed significantly higher ORO staining result when compared to other AVA-treat groups (AVA: Ctrl vs. S2, P<0.0001; AVA: S1 vs. S2, P<0.0001) (Fig 7E) indicating AVA does not significantly inhibit zebrafish Soat2. On the other hand, PPPA not only significantly decreased the intracellular neutral lipids in all groups Soat2-expressing cells compared to the vehicle control (Ctrl: DMSO vs. PPPA, P<0.001; S1: DMSO vs. PPPA, P<0.01; S2: DMSO vs. PPPA, P<0.0001), and the intracellular neutral lipids in control HEK293, Soat1- and Soat2-eGFP expressing cells are in comparable levels with the treatment of PPPA (Fig 7F) indicating that not only the endogenous human SOAT1 in HEK293 was inhibited, but also the zebrafish Soat2. Taken together, these results demonstrated that zebrafish Soat2 could catalyze the esterification of cholesterol and acyl-CoA and thereby increase the intracellular lipid droplets.

### Soat2 contributes to the yolk cholesterol consumption during zebrafish embryogenesis

**Fig 8:**
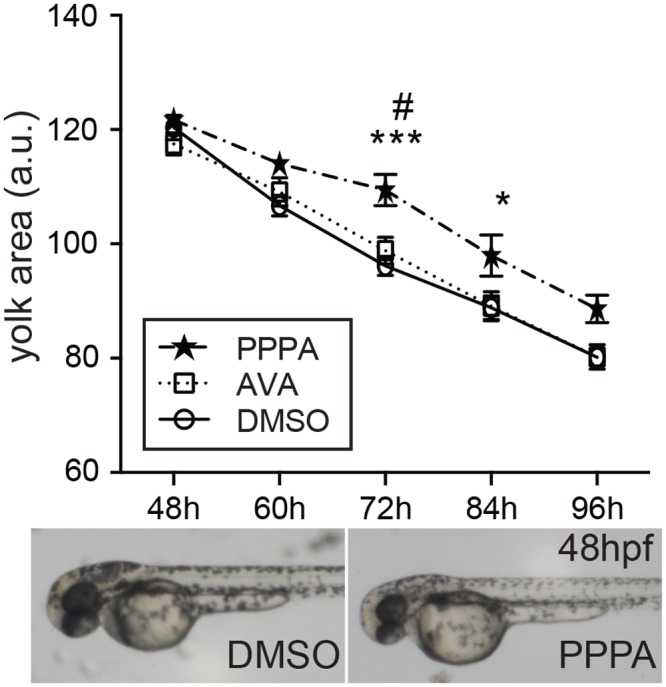
Delayed yolk consumption by yolk injection of PPPA. After the injection of PPPA, AVA or DMSO into the yolk at 3 hpf, embryos with grossly normal appearance at 24 hpf were subjected to yolk area measurement to estimate the yolk consumption. Yolk injection of PPPA resulted in significant larger yolk size at 72 and 84 hpf when compared to the DMSO group suggesting a delayed consumption of yolk content. *: P < 0.05, PPPA vs. DMSO; ***: P < 0.001, PPPA vs. DMSO; #: P < 0.05, PPPA vs. AVA

To test whether the enzymatic activity of Soat2 participates to the yolk cholesterol trafficking, we injected the Soat inhibitor PPPA or AVA into the yolk of 3 hpf zebrafish embryos and quantified their yolk size by using ImageJ software [24]. The yolk size begins to decrease dramatically after 48 hpf indicating rigorous yolk absorption by the embryo after this time point (Fig 8). Compared to the DMSO injected control embryos, the yolk size of the PPPA-treated group was significantly larger at 72 hpf (P = 0.0002, *n* = 27), and 84 hpf (P = 0.0343, *n* = 24) (Fig 8), suggesting that the loss of enzymatic activity of Soat2 in the YSL decreases the trafficking of yolk cholesterol towards embryo. Interestingly, the difference between PPPA treated embryos and the control embryos peaked at 72 hpf and this difference decreased and diminished at 96 hpf (Fig 8). On the other hand, embryos with AVA injected into the yolk showed no effect on the yolk size when compared to the control, while the yolk size of AVA-treated embryos was significantly smaller than PPPA-treated group at 72 hpf (Fig 8).

**Fig 9:**
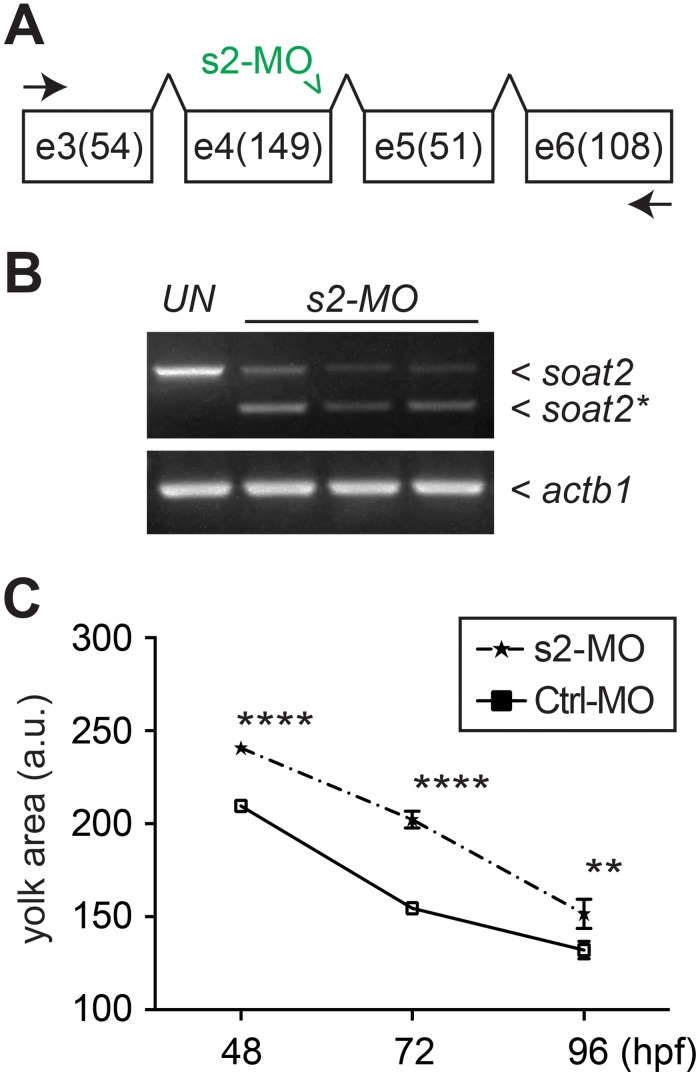
The yolk size phenotype in yolk-*soat2* knockdown zebrafish embryos. (A) The *soat*2 specific morpholino (s2-MO) was designed to target on the boundary of exon 4 and intron 4 (green concaved arrowhead). Specific primers for amplifying targeted sequence flank exon 3 through exon 6 (concaved arrows). (B) The effect of s2-MO was confirmed by reverse-transcription polymerase chain reaction (RT-PCR) and a shorter product (soat2*) was observed with the expected normal product (*soat2*) when s2-MO was injected to the embryos. The sequence of soat2* was confirmed to lack of the entire exon 4 and predictively to give rise to a truncated protein with only 99 amino acids. (C) The embryos with yolk injection of s2-MO at 3 hpf showed significantly larger yolk size when compared to the embryos received control morpholino (Ctrl-MO).

To validate that the yolk-hypoabsorption phenotype when treated with PPPA was due to the inhibition of Soat2, a morpholino oligo (s2-MO) against the boundary of exon 4 and intron 4 of *soat2* was obtained (Fig 9A). This morpholino oligo perturbs the maturation of *soat2* mRNA and in turn results in the omitting of entire exon 4 (soat2* in Fig 9B). This perturbed mRNA predictively introduces a frame-shift after exon 3 and gives rise to a truncated protein product (99 amino acids compared to 534 amino acids in an intact Soat2 protein). To specifically knockdown *soat2* in the yolk syncytial layer, s2-MO or control morpholino oligo (ctrl-MO) was injected into the yolk of 3 hpf embryos. Interestingly, the changes of the yolk sizes from 48 hpf to 96 hpf of *soat2* morphants and control embryos (Fig 9C) resembled the changes of PPPA and DMSO yolk-injected embryos (Fig 8). Moreover, the yolk sizes of the *soat2* morphants were significantly larger than those of control embryos (Fig 9C).

## Discussion

In this study, we identified 3 transcripts of *soat1* in zebrafish, and these transcripts contained a common short 5’-untranslated region (5’-UTR), identical coding sequence and three different 3’-UTRs. Accordingly, previous study revealed 4 different transcripts for human *Soat1* gene and three of them share a common short 5’-untranslated region and identical coding sequence, while the other transcript contains an additional long 5’UTR upstream from the exon 1 [31, 32]. This long 5′-UTR can impair the production of Soat1 protein by destabilizing its mRNA [33]. Although we did not find any transcripts with similar long 5′-UTR structure in zebrafish, it is possible that the post-transcriptional regulations of *soat1* may occur via the various 3’-UTRs.

There are controversial models of membrane topologies of Soat1 and Soat2. A 5-transmembrane domain model suggested that Soat1 and Soat2 share similar membrane structure with the catalytic activity positioned on opposite sides of the ER membrane [12]. The other model suggested that both enzymes bind fatty acid acyl-CoA at cytoplasm and catalyze the esterification in the ER membrane, despite the distinct membrane structures of the two enzymes [11, 34]. Due to the highly homologous sequences of zebrafish Soats to other vertebrate species, it would be interesting to see whether zebrafish Soat1 and Soat2 would fit in any of these two models.

Soats catalyze the esterification of cholesterol, which is generally believed to increase the intracellular lipid droplets along with other neutral lipids. Accordingly, we observed significantly increased neutral lipid (Fig 7A) and CE (Fig 7B) in the form of lipid droplets (Fig 7D and S2 Fig) in the cells overexpressing zebrafish Soat2. To our surprise, we observed neither increased neutral lipid (Fig 7A) nor accumulated CE (Fig 7B) in the cells overexpressing zebrafish Soat1 as compared with the control group. Accumulating studies in mice Soats led to the general belief that Soat1 predominantly contributes to the cellular cholesterol homeostasis whereas Soat2 plays its role in lipoprotein assembly for cholesterol absorption and transportation [35–38]. Furthermore, one study indicated that mammalian Soat1 and Soat2 responded differently to different types of fatty acid acyl-CoA [39]. Accordingly, our result suggested that these two paralogs might have distinct cellular functions. Alternatively, the stability of the mRNA or protein might also contribute to this result. In primates, the levels of *soat1* mRNA was much higher than that of *soat2* in hepatocytes, while Soat2 provided more than half of the total cholesterol-esterifying activity probably due to the distinct half-life of the proteins [40, 41]. In addition, our experiment was done in HEK293 cells, which also express human Soat1 [42]. It is also possible that the enzymatic activity of zebrafish Soat1 was either shadowed by the existence of human SOAT1 or too inefficient to be detected in HEK293 cells. The data reported in this study is insufficient to draw any conclusion on the catalytic characteristics of zebrafish Soat1. It may require a SOAT1-null cell line, such as AC29 [43], to elucidate the enzymatic activity of zebrafish Soat1.

As a Soat2 inhibitor, the IC_50_ of PPPA against SOAT1 (>20 to >100 μM) is much greater than it against SOAT2 (0.198~0.07 μM) [28, 44, 45]. In our study, we used a relatively high concentration (10 μg/mL or 17.135 μM). Accordingly, the cellular lipid accumulation was leveled by PPPA in Soat2-overexpressing cells compared to the wild-type HEK293 cells, while a general decrease was observed in PPPA treated cells when compared to the vehicle control (Fig 7F) indicating that not only the endogenous human SOAT1 in HEK293 was inhibited, but also the zebrafish Soat2. On average, the volume of a zebrafish embryo at 1-cell stage was assumed as 128 nL [46], while the calculation with our own measurement resulted in 143 nL. Accordingly, the working concentration of PPPA in the yolk-injection study could be assumed as 1.68~1.875 μg/mL (2.87~3.21 μM), a much lower dose than it was in the cell culture. Although we do not know whether the IC_50_ of PPPA against zebrafish Soat2 is similar to human SOAT2, this result supported the notion that zebrafish Soat2 has a conserved protein structure and enzymatic activity, and that zebrafish embryos could serve as a platform to study Soat2.

Similar to their distributions in the mammalians [10], we observed an ubiquitous expression profile of *soat1* with its mRNA detected as maternal message through 48 hpf and in every organ or tissue we examined in adult zebrafish (Fig 4). On the other hand, *soat2* was not detected until 12 hpf, and could only be found in the intestine, liver, brain and testis in the adult zebrafish (Fig 4). As our *in vivo* experiment with PPPA and morpholino oligos (Figs 8 and 9) indicated that the enzymatic activity of Soat2 at yolk syncytial layer indeed is important at 72 hpf, although the expression profiles implied that the existence of the Soat enzymes began at earlier stages.

**Fig 10:**
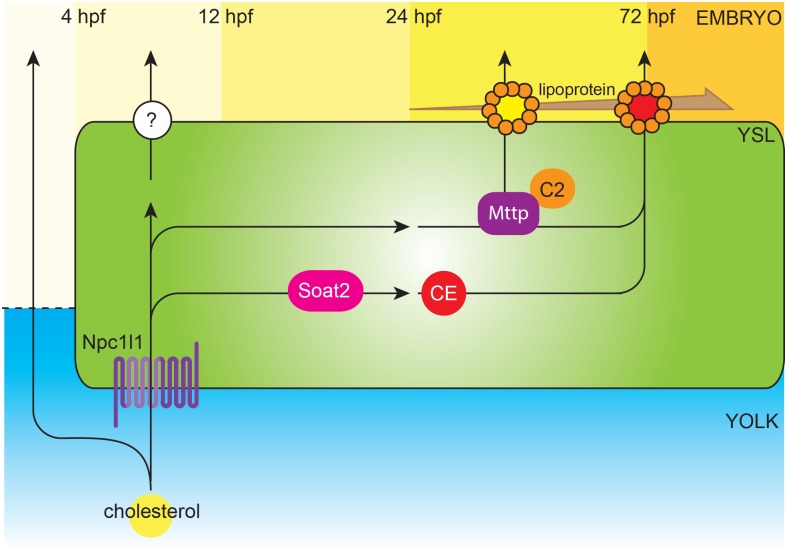
A schematic illustration of the molecular mechanism for yolk cholesterol trafficking in zebrafish embryo. Before the formation of YSL, free cholesterol diffuses from yolk to blastomeres directly. After YSL is formed at about 4 hpf, free cholesterol are transported from yolk to embryo through Npc1l1. The blood circulation in zebrafish embryos begins at about 24 hpf, and the importance of lipoproteins in yolk lipids absorption could be observed at 48 hpf when Mttp or Apo C-II was defective. The mRNA of *soat2* is expressed after 12 hpf, but its enzyme does not evidently contribute to the yolk cholesterol trafficking until the assembly of lipoproteins become prominent after 48 hpf. The level of CEs surged at 72 hpf and the loss of Soat2 activity resulted in delayed yolk consumption most prominently at 72 hpf indicated that the activity of Soat2 peaked at 72 hpf.

Previous studies indicated that the expression of cholesterol transporter Niemann-Pick C1-Like 1 (*npc1l1*) at yolk syncytial layer is critical for the cellular movement of epiboly during gastrulation in zebrafish embryos [47]. Furthermore, zebrafish genes related to the apolipoprotein packaging such as Microsomal triglyceride transfer protein (*mttp*) and Apolipoprotein C-II (*apoc2*) play an important role in yolk lipid trafficking: the yolk consumption was markedly reduced after 48 hpf when these genes were knocked-down [19, 20]. Our study reported a significant reduction in yolk consumption at 72 and 84 hpf when enzymatic activity of Soat was inhibited by PPPA, while morpholino oligo guided *soat2* knockdown resulted in delayed yolk absorption at 48 hpf through 96 hpf with the same trend that the difference between *soat2* inhibited group and control group was most prominent at 72 hpf. In accordance, previous studies in avian embryos suggested the CEs at yolk sac become predominant at the near-hatching stage [48, 49]. Notably, a recent study indicated that yolk fatty acids were not actively contributing to CE synthesis until 72 hpf and this contribution could be inhibited by Soat inhibitor [50]. These results and ours indicated that the content and the mechanisms of cholesterol trafficking from the yolk to the embryo may vary along the developmental progress (Fig 10) and the nutrient requirement of the embryos might be very different between initial and later stages of embryogenesis. Since it is believed that the esterification of cholesterol increases the trafficking efficiency, it is reasonable to speculate that the lipid trafficking efficiency increases as the embryonic development progress due to the growth of the energy and nutrient demand of the embryo. Therefore, it is not surprising to observe the enzymatic activity of Soats peaked as the embryos approaching near the hatching stage.

Earlier attempts to use non-selective SOAT inhibitors such as AVA failed to prevent atherosclerosis [51]. Recent studies indicated that hepatic or intestinal SOAT2 might play a more important role in hypercholesterolemia or atherosclerosis [52, 53]. Accordingly, the studies on SOAT2-specific inhibitors, such as PPPA, revealed the potential of this class of compounds to treat hypercholesterolemia and atherosclerosis [54, 55]. In line with these findings, our results indicated that AVA treatment in the yolk did not, while PPPA treatment did, hindered the yolk consumption measurably in morphometry (Fig 8). For this experiment, 1.4 ng of AVA was injected into each treated embryo. Accordingly, the working concentration of AVA in the yolk-injection study could be assumed as 9.79~10.9 μg/mL, which is more than 10 times exceeding the necessary concentration for cell culture experiment. Therefore, this result of AVA was unlikely due to an insufficient dose of AVA. Furthermore, the yolk cholesterol trafficking mechanisms in zebrafish embryos (Fig 10), as revealed here and by other groups [19, 20, 47], are similar to that in mammalian intestine. These findings indicated that zebrafish embryo could serve as a potential and promising platform to study lipid ingestion and Soat2 inhibitor screening.

In conclusion, zebrafish Soat1 and Soat2 were highly conserved throughout the evolution. *Soat1* mRNA was ubiquitously expressed temporally and spatially, while the s*oat2* mRNA was not detected until 12 hpf and expressed in a tissue specific manner in the adult zebrafish. *Soat2* was detected in the yolk syncytial layer, hatching gland, developing cardiovascular system and gastrointestinal tract during the embryonic development. We demonstrated the enzymatic activity of Soat2, which can be inhibited by PPPA, and the knockdown or inhibition of this enzyme resulted in a slower rate of yolk cholesterol consumption indicating that Soat2 contributed to the yolk cholesterol trafficking during embryogenesis.

## Supporting Information

S1 Fig3’-RACE resulted in 3 variants in 24 hpf zebrafish *soat1* transcripts.(A) A 3’-RACE was performed with a reaction with no template as negative control. Multiple significant products from nested-PCR could be seen (Sample). After cloning and sequencing, three variants were confirmed (soat1 3’-RACE #1, #2 and #3). (B) BLAT search was performed and located the three 3’-RACE results in the genome browser. The sequence of *soat1* 3’-RACE #1 is generally identical to transcript *soat1-001*, while the other two results are not in the genome browser. The sequences of the three 3’-RACE results were as shown below and the font colors match to the relative genomic locations marked here.***soat1* 3’-RACE #1** (**518 bp**)$\underline{\text{GGACAGGGGGTAATGATCT}}\text{GCCTGTATTCTCAAGAGTGGTACGCACAACGCTACTGTCCCATTGCAGAG}\text{CCTTCCTTGATTGACCTGCTGAAGCCTCGATCCTGGACTTGTTATCCACAGACTAACGCTGCTGTTGACTCTCACTGAATGGGATTAACACAGAAGAACATTCTTTAG}\text{GTATTGCACTATGAGTGAAAAATGAAAATCACGAAGACAACGTTTTATGAAGGGGCCTTAGATATGTTATTTGTTGAGATATTATTTATTATGACAGCCGTAACAGAATGTGGAACGTCTATAATGTTTCAATAGTTTAGTCTAGTTAGCATTTGACAATCTATTTTAGGTTCAATCTGATCTGGCTTAAAGAGATTCTATTTTTAGACGCCTTTGTGATGTCATGAAGTTGAGATCAGATTTAGTTTACTGCCGTTTTATTTAGATTTTTAGTGTTTGATACTGTTGTGTTATTCCATGGACTTAAAGAGTGAATATGTGGTA}\text{AAAAAAAAAAAAAAAAA}$***soat1* 3’-RACE #2** (**574 bp**)$\underline{\text{GGACAGGGGGTAATGATCT}}\text{GCCTGTATTCTCAAGAGTGGTACGCACAACGCTACTGTCCCATTGCAGAG}\text{CCTTCCTTGATTGACCTGCTGAAGCCTCGATCCTGGACTTGTTATCCACAGACTAACGCTGCTGTTGACTCTCACTGAATGGGATTAACACAGAAGAACATTCTTTAG}\text{TGTCCTAGAGATGGGTTGCGGCTGGAAGGGCATCCGCTCCGTAAAAACACGCTGGATAAGTTGGCGGTTCATTCCGCTGTGGCGACCCCAGATTAATAAAGGGACTAAGCCGACAAGAAAATGAATGAATGAATGAGTATTCACATTAGCACAGCTTTTATGACATCTAATATATTCGTTAAAATGACATTGTTAGGCCTATGTAACTTAAGCGTTCTTAGCAGACACAACTTTTTATCTGTTTTGGGCTAATTGTAGGCTACGTTGGTTATCATCCGTAAGATGTTATTTGCTGTACTTTATGAATGTACATTTTCTCTTAGTTGTGCTTATGTTATTATTAATGTATTAAAAAGAGCGATGTTTTGCATCACGGTGA}\text{AAAAAAAAAAAAAAAAAA}$***soat1* 3’-RACE #3** (**1655 bp**)$\underline{\text{GGACAGGGGGTAATGATCT}}\text{GCCTGTATTCTCAAGAGTGGTACGCACAACGCTACTGTCCCATTGCAGAG}\text{CCTTCCTTGATTGACCTGCTGAAGCCTCGATCCTGGACTTGTTATCCACAGACTAACGCTGCTGTTGACTCTCACTGAATGGGATTAACACAGAAGAACATTCTTTAG}\text{GTATTGCACTATGAGTGAAAAATGAAAATCACGAAGACAACGTTTTATGAAGGGGCCTTAGATATGTTATTTGTTGAGATATTATTTATTATGACAGCCGTAACAGAATGTGGAACGTCTATAATGTTTCAATAGTTTAGTCTAGTTAGCATTTGACAATCTATTTTAGGTTCAATCTGATCTGGCTTAAAGAGATTCTATTTTTAGACGCCTTTGTGATGTCATGAAGTTGAGATCAGATTTAGTTTACTGCCGTTTTATTTAGATTTTTAGTGTTTGATACTGTTGTGTTATTCCATGGACTTAAAGAGTGAATATGTGGTATATTTTGGCTGTTTTTCTGCCTGGACCACAAAAAAAATCTAAAATGTTTACAAATGGGGTTTTGAAATCTAAAACCCCAAAGGAAACAATGTGCCATACTGCTGTGAAACATTCCTTCATTCATTTTTTTCGGCATGGTCCCTTTATTCATCTGGGTTTCTGGTTTCTGGGTTTCTGGAAGCAGATTCTAGCAGCTGAGGGCGTAGTAGCTGAAAGCCCATTCACCCTGCTTTGACTGAACTCTTGGAGCTTCTAATTTATTTGATCCTAAA}\text{TCTGAGCGAGCATATCTGTCATGTATTGAGTCCTAGCCATTTAGCATTTATAGACAGTAATATACTTTAAATCTATTCTGATGTACTGGGAGCAGTGTAAAGACTGAGACAG}\text{TATGATCTGCTGTGATTTCCTGGTTTTGGTCAGAATCCTGGCAGCAGCGTTCTGGATGAGCTGTAACTGTCTGAATGTCTTTTTGGGAAGGCCTGTGAGGAGTCTATTACAGTAATCCACCCTGCTGCTGATAAAAGCATGATCAAGTTTCTCTAAGTCCTCACTGGGAACAAAGCTTCTGATTCTTGCAATGTTTTTGAGATGATAGTATGCTGATTTACTGACTGCTTTGACATGCTTTAACTCATGACTTTGACATGACTACACTGGGAAACATCCATACACACTCAGTCACACATACACTAAGTACAATTTAGGTTATTCAATTCACCTATAACACATGTCTTTGAACTGTGGGAGAAACCAGAGCACCCAGAGGAAACCCATGCGAACACGGGGAGAACATGCAAACTCCACACAGAAATGTCAACTGACCCAGCCTGGACTCGAACCAGCAACATTCTTGCTGTGAGGCAACACTGCTAACCACTGAGCCATCGTGTCACCCTTGTGAAACATAAAAACTCATTTAATTCTAGAGCTTTAATTTGGAGGAAGTTCTCCGTTCTGCTCATTTGTTCTCTTTGGTTACTGTTTGAAGAGTCTGGTTCACACCAGGAGTGAAAATAGGACTATATTATCAACTCATAATAGCTGGGTTTCCATCATCACATGTTAATGCACATTTTGAAGTATCGCATGGGAAACGAGTGATGCCAAATGATGAATAAAACTAGTTTTTACACTC}\text{AAAAAAAAAAAAAAAAAAAAAA}$(TIF)

S2 Fig Zebrafish Soat2 catalyzes the esterification of cholesterol. After 2- and 5-hour incubations with 150 μM oleic acids, 10 μg/mL cholesterol and 10 μg/mL NDB-cholesterol, the intracellular accumulation of CEs was observed in the HEK293 cells transiently overexpressing DsRed, zebrafish Soat1-DsRed and zebrafish Soat2-DsRed. The progress of CE accumulation could be seen in cells with zebrafish Soat2 overexpression.
(TIF)
