# Supplementary figures and images for: Sterol O-Acyltransferase 2 Contributes to the Yolk Cholesterol Trafficking during Zebrafish Embryogenesis
Source: PLoS One. 2016 Dec 9;11(12):e0167644. doi: 10.1371/journal.pone.0167644 (PMC5147938; doi:10.1371/journal.pone.0167644)

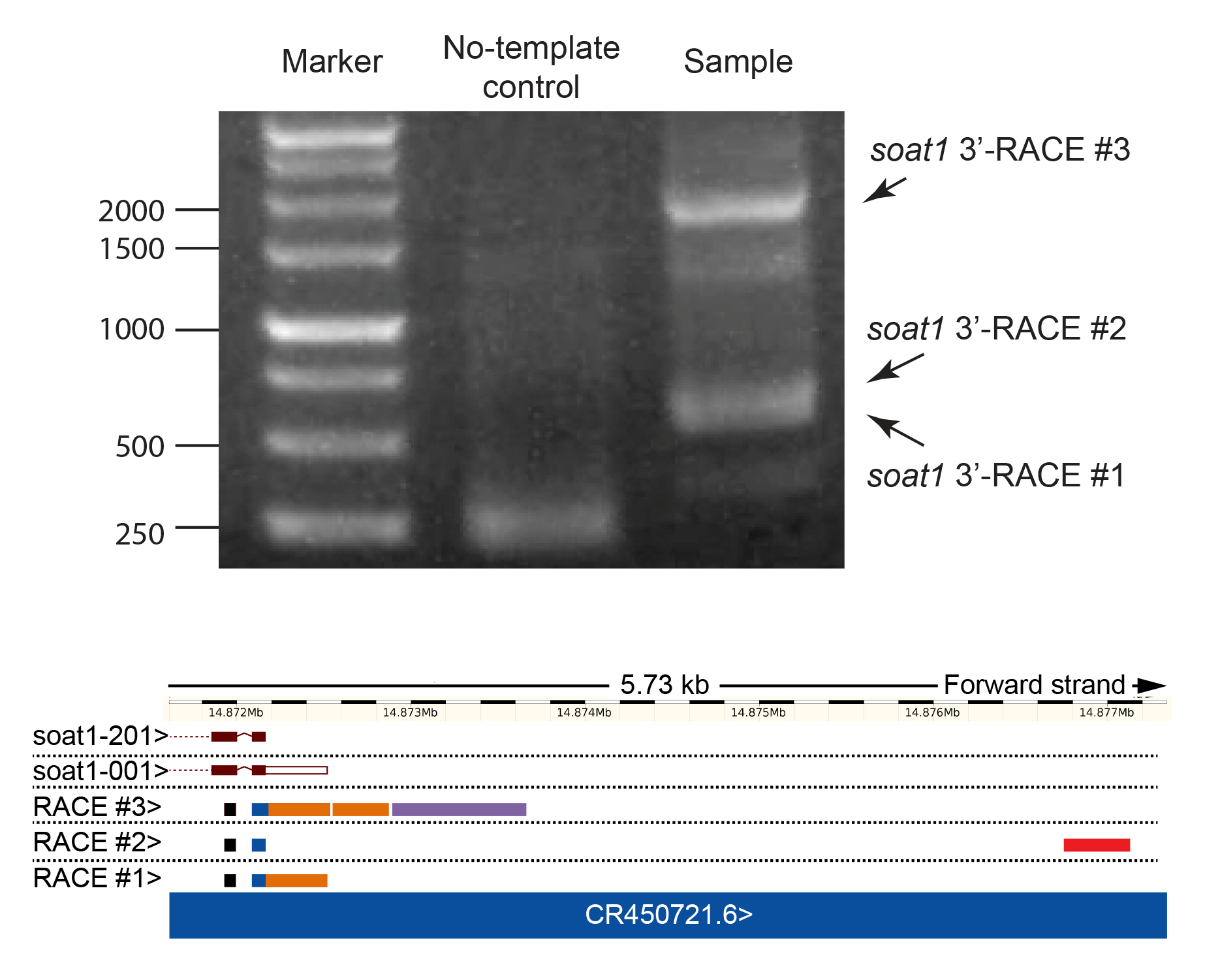

Supplement: S1 Fig — (A) A 3’-RACE was performed with a reaction with no template as negative control. Multiple significant products from nested-PCR could be seen (Sample). After cloning and sequencing, three variants were confirmed (soat1 3’-RACE #1, #2 and #3). (B) BLAT search was performed and located the three 3’-RACE results in the genome browser. The sequence of soat1 3’-RACE #1 is generally identical to transcript soat1-001, while the other two results are not in the genome browser. The sequences of the three 3’-RACE results were as shown below and the font colors match to the relative genomic locations marked here. soat1 3’-RACE #1 (518 bp) GGACAGGGGGTAATGATCT_GCCTGTATTCTCAAGAGTGGTACGCACAACGCTACTGTCCCATTGCAGAGCCTTCCTTGATTGACCTGCTGAAGCCTCGATCCTGGACTTGTTATCCACAGACTAACGCTGCTGTTGACTCTCACTGAATGGGATTAACACAGAAGAACATTCTTTAGGTATTGCACTATGAGTGAAAAATGAAAATCACGAAGACAACGTTTTATGAAGGGGCCTTAGATATGTTATTTGTTGAGATATTATTTATTATGACAGCCGTAACAGAATGTGGAACGTCTATAATGTTTCAATAGTTTAGTCTAGTTAGCATTTGACAATCTATTTTAGGTTCAATCTGATCTGGCTTAAAGAGATTCTATTTTTAGACGCCTTTGTGATGTCATGAAGTTGAGATCAGATTTAGTTTACTGCCGTTTTATTTAGATTTTTAGTGTTTGATACTGTTGTGTTATTCCATGGACTTAAAGAGTGAATATGTGGTAAAAAAAAAAAAAAAAAA soat1 3’-RACE #2 (574 bp) GGACAGGGGGTAATGATCT_GCCTGTATTCTCAAGAGTGGTACGCACAACGCTACTGTCCCATTGCAGAGCCTTCCTTGATTGACCTGCTGAAGCCTCGATCCTGGACTTGTTATCCACAGACTAACGCTGCTGTTGACTCTCACTGAATGGGATTAACACAGAAGAACATTCTTTAGTGTCCTAGAGATGGGTTGCGGCTGGAAGGGCATCCGCTCCGTAAAAACACGCTGGATAAGTTGGCGGTTCATTCCGCTGTGGCGACCCCAGATTAATAAAGGGACTAAGCCGACAAGAAAATGAATGAATGAATGAGTATTCACATTAGCACAGCTTTTATGACATCTAATATATTCGTTAAAATGACATTGTTAGGCCTATGTAACTTAAGCGTTCTTAGCAGACACAACTTTTTATCTGTTTTGGGCTAATTGTAGGCTACGTTGGTTATCATCCGTAAGATGTTATTTGCTGTACTTTATGAATGTACATTTTCTCTTAGTTGTGCTTATGTTATTATTAATGTATTAAAAAGAGCGATGTTTTGCATCACGGTGAAAAAAAAAAAAAAAAAAA soat1 3’-RACE #3 (1655 bp) GGACAGGGGGTAATGATCT_GCCTGTATTCTCAAGAGTGGTACGCACAACGCTACTGTCCCATTGCAGAGCCTTCCTTGATTGACCTGCTGAAGCCTCGATCCTGGACTTGTTATCCACAGACTAACGCTGCTGTTGACTCTCACTGAATGGGATTAACACAGAAGAACATTCTTTAGGTATTGCACTATGAGTGAAAAATGAAAATCACGAAGA [file pone.0167644.s001.tif]

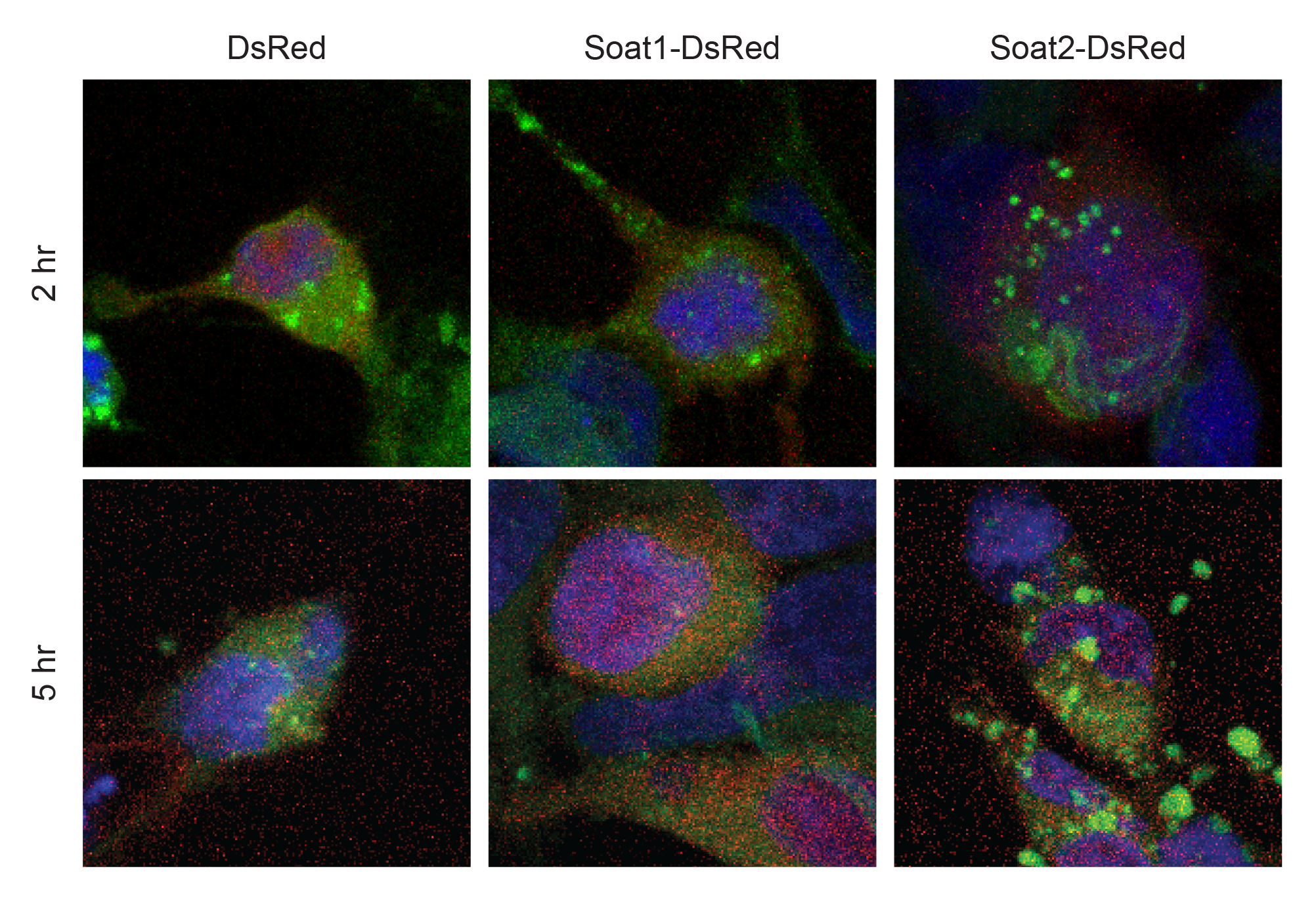

Supplement: S2 Fig — After 2- and 5-hour incubations with 150 μM oleic acids, 10 μg/mL cholesterol and 10 μg/mL NDB-cholesterol, the intracellular accumulation of CEs was observed in the HEK293 cells transiently overexpressing DsRed, zebrafish Soat1-DsRed and zebrafish Soat2-DsRed. The progress of CE accumulation could be seen in cells with zebrafish Soat2 overexpression. (TIF) [file pone.0167644.s002.tif]
